# Open-label trial with artemether-lumefantrine against uncomplicated *Plasmodium falciparum* malaria three years after its broad introduction in Jimma Zone, Ethiopia

**DOI:** 10.1186/1475-2875-11-240

**Published:** 2012-07-23

**Authors:** Teferi Eshetu, Nasir Abdo, Kunuz H Bedru, Sintayehu Fekadu, Andreas Wieser, Michael Pritsch, Thomas Löscher, Nicole Berens-Riha

**Affiliations:** 1 Division of Infectious Diseases and Tropical Medicine, Medical Center of the University of Munich (LMU), Leopoldstrasse 5, 80802, Munich, Germany; 2 Department of Medical Laboratory Sciences and Pathology, Jimma University (JU), Jimma, Ethiopia; 3 Jimma Zone Health Bureau, Jimma, Ethiopia; 4 Department of Internal Medicine, Jimma University (JU), Jimma, Ethiopia; 5 Max von Pettenkofer-Institute of Hygiene and Medical Microbiology, Munich, Germany; 6 German Centre for Infection Research (DZIF) at LMU, Munich, Germany

**Keywords:** Malaria, *Plasmodium falciparum*, Artemether-Lumefantrine, ACT, Ethiopia

## Abstract

**Background:**

In Jimma Zone, Ethiopia, the first-line treatment of uncomplicated falciparum malaria has been changed from sulphadoxine-pyrimethamine (SP) to artemether-lumefantrine (AL) in 2006. The objective of this study was to assess the effectiveness of AL in Jimma Zone two to three years after its broad introduction.

**Methods:**

An open-label, single-arm, 42-day study of AL against falciparum malaria was conducted in four areas with moderate transmission in Jimma Zone between November 2008 and January 2009 and between August and December 2009. Patients (one-81 years) with uncomplicated *Plasmodium falciparum* mono-infection were consecutively enrolled. Follow-up visits were at day 2, 3, 7, 28 and 42 or any other day if symptoms reoccurred. Primary and secondary endpoints were PCR-corrected and uncorrected cure rates (molecular differentiation between recrudescence and re-infection) on days 28 and 42. Other secondary endpoints were gametocytaemia at day 7 and day 28, parasitaemia at day 2 and 3, and re-infection rates at day 28 and day 42.

**Results:**

Of 348 enrolled patients, 313 and 301 completed follow-up at day 28 and at day 42, respectively. No early treatment failure occurred. For per protocol analysis, PCR-uncorrected cure rates at day 28 and 42 were 99.1% (95% CI 98.0-100.0) and 91.1% (95% CI 87.9-94.3), respectively. PCR-corrected cure rates at day 28 and 42 were 99.4% (95% CI 98.5-100.0) and 94.7% (95% CI 92.2-97.2), respectively. PCR-corrected cure rate at day 42 for children ≤5 years was 90.6% (95% CI 82.4-98.7) only. Adverse events were in general mild to moderate. Incidence of new infections was 3.4% during 42 days, no new infections with *Plasmodium vivax* were observed. Microscopically detected gametocytaemia was reduced by 80% between day 0 and day 7.

**Conclusion:**

In general, AL was effective and well tolerated in Jimma Zone, Ethiopia. However, the PCR-corrected recrudescence rate per-protocol at day 42 for children ≤5 years was 9.4%. Therefore, further development should be monitored on a regular basis as recommended by WHO.

## Background

Due to increasing resistance of *Plasmodium falciparum* against previous anti-malarials, such as chloroquine and sulphadoxine-pyrimethamine (SP), most African countries, including Ethiopia, have changed their national policy in recent years towards first-line treatment with artemisinin-based combination therapy (ACT) [1,2]. ACT produces faster relief of clinical symptoms and parasite clearance in uncomplicated *P. falciparum* malaria than any other currently available anti-malarial drug [3,4]. It combines the potential of rapid reduction of the parasite burden with the elimination of remaining parasites due to longer acting partner drugs [5]. ACT seems to be well tolerated as several studies indicate; no severe side effects have been observed, especially for the combination artemether-lumefantrine (AL) [6,7].

Malaria is endemic in approximately 75% of the national territory of Ethiopia. About 50 million inhabitants are at risk of infection. In 2009, there was a considerable increase of malaria-associated morbidity and mortality in the south and south-west of Ethiopia, including the town of Jimma and its surroundings, with most cases occurring from September to December. Predominant species was *P. falciparum*, but a trend to mixed and mono-infections with *Plasmodium vivax* was observed [8,9], pers comm with local health centres]. AL was introduced in Ethiopia as first-line treatment for uncomplicated falciparum malaria in 2004 and became available in Jimma Zone in 2006. Since then, AL was provided free of charge at public health centres by the government only.

Increasing recrudescence rates and reduced *in vitro* response to artemisinins were first reported from some Asian countries [10], where artesunate monotherapy has commonly been used for some decades. Along with sub-therapeutic doses, incomplete treatment courses, substandard and fake artemisinin-based drugs, this constitutes a major risk factor for the development of drug resistance. In 2008, the alarming news of delayed parasite clearance rates after treatment with artesunate monotherapy in Cambodia arrived. Reports on increasing recrudescence and late clearance rates with ACT followed [11-14]. A new definition for artemisinin resistance was introduced by the WHO [10]. Interestingly, slow clearance rates did not seem to be associated with decreased sensitivity to drugs *in vitro,* but were associated with parasite genetics in Cambodia [15]. Resistance spread on a genetic basis would enormously compromise the recent success of ACT in reducing the incidence of malaria in various regions [16,17]. Since the WHO memorandum in 2011, the use of artemisinin-based monotherapies is no longer recommended [18].

The broad introduction of ACT in many African countries offers the chance of monitoring drug-resistance development right from the start. Several studies from neighbouring countries and from Ethiopia showed high cure rates for AL and other ACT [19-26]. However, one recent study raised concerns over prolonged clearance rates in Africa [27].

The underlying study was aimed at investigating the clinical and parasitological effectiveness of AL in Jimma Zone two to three years after its broad introduction.

## Methods

### Study area and recruitment

Jimma is the capital of Jimma Zone in Oromiya regional state and is located 355 km south-west of the Ethiopian capital, Addis Ababa. Jimma Zone and its surroundings are malaria endemic with a low to moderate transmission. The predominant species in most areas is *P. falciparum*, followed by *P. vivax*. Most cases occur from September to December after the main rainy season from June to August. Recruitment took place over eight months between November 2008 to January 2009 and August 2009 to December 2009. Study sites were Agaro Health Centre, Jimma Health Centre, Serbo Health Centre, and Asendabo Health Centre (average elevation of study areas between 1,667 m to 1,772 m above sea level).

### Study design and patients

The study was an open-label, single-arm, non-supervised trial with standard treatment artemether-lumefantrine in a six-dose, weight-adapted regimen. The study was implemented in routine diagnostic of the health centres. Blood slides were performed from all patients with fever. Only falciparum mono-infection was considered for the study. After microscopic confirmation, patients were informed about the study and screened for eligibility if consent was given.

Inclusion criteria were age above one year, body weight above 5 kg, uncomplicated *P. falciparum* malaria (mono-infection with *P. falciparum*), parasite density 1,000–100,000/μL, axillary temperature ≥37.5°C or recorded history of fever within preceding 24 hrs, ability to tolerate oral therapy, informed consent by the patient or the legal representative as well as residency in the study area. Treatment of uncomplicated malaria is on an outpatient basis, therefore, eligibility for the study was limited to residency in walking distance to the health posts or the ability to use public transportation (<2 hours of travel time) due to the follow-up schedule of the study.

Exclusion criteria were any anti-malarial treatment within the previous seven days (anti-malarials like chloroquine, AL, SP, quinine and others as well as antibiotics with anti-malarial effects like doxycycline, clindamycine), mixed plasmodial infection, danger signs (inability to drink, repeated vomiting, recent history of convulsions, lethargy or unconsciousness) and signs of severe malaria as defined by the WHO [28] or any other known severe underlying disease (eg, cardiac, renal, hepatic diseases, severe malnutrition, known HIV infection). Patients with history of allergy or intolerance against study medications, during pregnancy or lactation were also excluded.

### Ethical considerations

Each patient (or parents/guardians) received oral and written information about the study and signed an informed consent form. Ethical approval was obtained from the Ethical Board of Jimma University, the Ethiopian Federal Ministry of Science and Technology, and from the Ethical Committee of the Faculty of Medicine of the Ludwig-Maximilian-University (LMU) in Germany.

### Treatment

The standard oral regimen of Coartem® (Novartis Pharmaceuticals Corporation, Suffern, New York, USA for Novartis AG, 20 mg artemether/120 mg lumefantrine) was used for treatment which is supplied free of charge by the government at health centres and hospitals. The government is the only legal distributor of AL in Ethiopia. In a drug quality control centre in Addis Ababa, drug tests are performed on a regular basis to exclude fake drugs.

AL was administered as follows: 20 mg of artemether and 120 mg of lumefantrine (children 5–14 kg), 40 mg/240 mg (children 15–24 kg), 60 mg/360 mg (children 25–34 kg), 80 mg/480 mg (adults and children ≥35 kg bwt.), at hrs 0, 8, 24, 36, 48 and 60 (six doses). Patients were instructed to take the doses with fatty food. Full treatment was supplied on admission. The first dose was administered at the health centres by medical staff under supervision. Full re-dosing was performed, if vomiting occurred within 30 mins after the first dose given. If a patient was unable to tolerate the medication, treatment was discontinued and the patient referred to the hospital for intravenous (IV) treatment with quinine. Therapy for early treatment failures would have followed the respective local guidelines for second-line therapy and consisted of IV quinine. Late treatment failures were treated with AL.

### Examinations and follow-up procedures

Upon initial examination, a rapid screening procedure was established. Blood was generally taken by finger-prick method. Blood slides were prepared at initial presentation for all patients with fever or history of fever within 24 hrs, during routine examination at all study centres. Sampling and preparation of thin and thick smears was performed at each study centre by physicians and laboratory technicians, who had been trained and instructed by investigators at beginning of the study. Microscopic assessment of Giemsa-stained thin and thick blood smears confirmed *P. falciparum* mono-infection.

A general physical examination was performed on admission. After recruitment, patients were questioned via checklist about age, area of residency, underlying diseases, symptoms, with special emphasis on danger signs and symptoms for severe malaria and medications in the previous three weeks. If included, the treatment was given under observation and an additional blood sample was spotted on filter paper (Whatman 3MM) (day 0).

Patients returned for outpatient follow-up on day 2, 3, 7, 28 and 42 (+/−7) and on any day before day 42 if any symptoms recurred. Day 1 was not a regular study day although patients were instructed to return immediately if symptoms worsened. Each patient was asked on every visit about past (since last visit) and present symptoms, every adverse event was documented in specific case report forms and later transferred to the database. Severe adverse events had to be reported within 24 hours to the study PI and the ethical boards. During follow-up, thin and thick blood smears and blood-spotted filter papers were simultaneously prepared on every visit for further molecular investigations.

### Endpoints

Primary and secondary endpoints were PCR-corrected and uncorrected cure rates on days 28 and 42. Further endpoints were treatment failures, completion of the follow-up period without treatment failure meaning adequate clinical and parasitological response (ACPR), loss to follow-up, voluntary and involuntary withdrawal from study and protocol violation. Treatment failures were categorized as early treatment failure (ETF), late clinical failure (LCF), and late parasitological failure (LPF) according to the definition of treatment failures for low to moderate transmission areas determined in the WHO protocol issued in 2006. Other secondary endpoints were defined as gametocytaemia at day 7 and day 28, parasitaemia at day 2 and day 3, and PCR-corrected re-infection rates at day 28 and day 42.

### *In vivo* resistance definition and clearance time calculation

Prolonged parasite presence (parasitaemia at day 3 ≡ ≥72 hours after treatment start) in ≥10% of cases after treatment with an ACT is defined as suspected resistance according to WHO [10], although this statement seems to need further evaluation. Artemisinin resistance seems to be highly unlikely if ≤3% patients show parasitaemia at day 3, if initial parasite density was between 10,000-100,000/μL [29].

The formula for parasite clearance was derived from the formula of half-life time: e^- λt^.

λ = ln(0.5), constant of decay. P_2_ = parasitaemia at day 2, P_0_ = parasitaemia at day 0.

P_2_ = P_0_ (e^(−ln(0.5)/-t^_2_^)(48)^) Correlating with t_2_ = (ln(0.5)/ln(P_0_/P_2_)) (−48 hrs), for calculation of half-life time t_2_ between day 0 and day 2, meaning the time half the parasites were cleared every t_2_ during 48 hrs. Analogue parasitaemia at day 3 and t_3_.

### Laboratory examinations

Thin and thick blood smears were prepared according to standard procedures. Slides were stained with 10% Giemsa for 20 mins and independently read by two microscopists. Parasite density was counted based on standard procedures. The number of asexual parasites was counted against 200 leukocytes assuming 8,000 leukocytes per μL. Smears were determined to be negative only after examining 120 fields [100x ocular]. Parasite densities differing by more than 10% between microscopists were controlled by a third microscopist, whose result was final.

DNA from dried blood spots on Whatman filter paper (3MM) was extracted by Chelex method as described elsewhere [30]. DNA from all samples from day 0, day 42 and from all treatment failures (recurrent parasitaemia) was extracted.

Molecular genotyping was performed by polymerase chain reaction (PCR) from all samples from day 0 and all treatment failures. Therefore, sequences of parasite genes coding for the polymorphic proteins glutamate-rich protein (GLURP) and merozoite surface proteins 1 and 2 (MSP-1 and MSP-2) were amplified by nested PCR assays. *Plasmodium falciparum* isolates were obtained at day 0 and any day of possible treatment failure up to day 42 of follow-up. PCR products of the paired samples were compared. If any pre-treatment alleles differed from post-treatment alleles, a new infection was considered; if no new allele in pre- and post-treatment samples occurred, a recrudescence was assumed [31,32].

Species amplification was performed by conventional nested PCR described by Snounou [33]. The species from all samples from day 0, day 42 and from all treatment failures was determined.

For detection of mature gametocytes, the amplification of *Pfs25* mRNA by real-time quantitative nucleic acid sequence based amplification (QT-NASBA) was chosen as target sequence as described elsewhere [34,35]. RNA extraction was performed with using NucliSens®- easyMAG® Lysis Buffer. Filter paper was cut out and rocked at 150 rpm for 30 mins at room temperature, following the original RNA extraction method described by Boom and colleagues [36]. The solution was centrifuged at 1,500 g for 5 mins, the filter paper removed and 50 μl of silica solution was added to the solution. The extraction was made with the NucliSens®- mimiMAG ™ unit (bioMérieux SA, Lyon, France) according to the manufacturer’s manual. Amplification was performed using NucliSENS EasyQ® Basic Kit (bioMérieux Bv, RM Boxtel, The Netherlands) according to the manufacturer’s manual and as described recently [35]. Microscopically gametocyte-positive and a random selection of 10% of microscopically gametocyte-negative samples were chosen.

Filter paper samples were stored at room temperature until transport to Germany (one to 12 months) and then stored at −20°C. DNA extraction was performed after arrival of the samples in Germany. RNA extraction for QT-NASBA was conducted about 18 months later.

### Data management and statistical analysis

Data forms were checked regularly by the primary study coordinator at the site. Double data entry in Excel (Microsoft Office 2007) was independently performed in Ethiopia and Germany. All data are subject to the data protection and were processed anonymously. Data were transferred to STATA, version 11, (Statacorp, 2009) and analysis was performed. The traditional “per-protocol” and “intention-to-treat” approach were applied. Logistic regression models were used for a multivariate analysis of outcome variables (primary and secondary endpoints) and independent variables (age, gender, site, season, weight, height). Comparison between two groups was calculated by the Student’s *t*-test or Wilcoxon rank sum test for continuous variables and the *χ*^2^ test or Fisher’s exact test for categorical variables. Results were considered significant if p-values were <0.05.

The anticipated population proportion of clinical and parasitological failures was estimated based upon the data of a study at Jimma Hospital in 2006 [37]. The failure rate in a 42-day course was 0%; one symptomatic parasitaemia occurred on day 70 that seemed to be a recrudescence. Compared with other studies showing failure rates of about 5%, a failure rate of maximum 10% was chosen [10]. Assuming follow-up losses and withdrawals during a follow-up period longer than 14 days of 20% and ACPR of 90%, a sample size of n = 400 was chosen to result in a confidence interval from 86.71-93.29% on a 95% confidence level. Due to screening failures, the resulting confidence interval was 86.68%-93.32%.

## Results

### Baseline data

**Figure 1:**
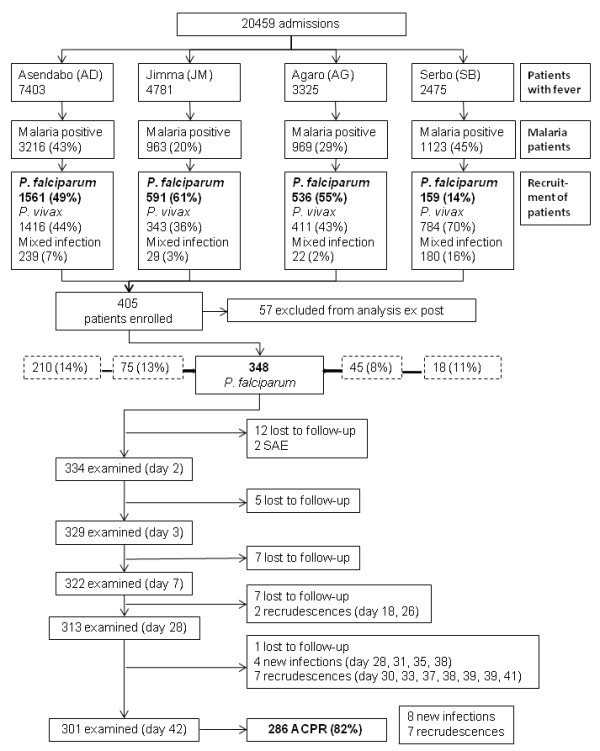
Flowchart

The study was implemented in routine diagnostic of the outpatient service (Figure 1). From 20,459 patients with fever, thin and thick smears were taken for malaria diagnosis (official numbers from the health centres for the study period). Thirty-one percent (range 20-45%) had malaria among these were 2,847 *P. falciparum* mono-infections. Treatment of uncomplicated malaria is on an outpatient basis. Eligibility for the study was limited to residency near to the health posts due to the follow-up schedule of the study. A total of 405 patients with uncomplicated *P. falciparum* malaria were finally enrolled from four different sites in Jimma Zone between November 2008 and January 2009 and from August to December 2009. The study was planned to be completed during the first sampling period 2008/2009 but sample collection had to be extended until end of 2009 due to weather conditions and logistics. Only 23% were recruited during the first season. Reasons for non-eligibility were age below one year, underlying chronic diseases, refusal, pregnancy, off-limits parasitaemia and in over 90% residency too far away from the health centres.

Sixty percent of the patients were from Asendabo, 22% from Jimma, 13% from Agaro and 5% from Serbo (Table 1); 60% were male; mean age was 17 years (95% CI 15.7-18.5, range 1–81). Half of the patients were under 16 years old, only 13% over 50. Children between one and five years were equally distributed between sites. Mean parasitaemia was significantly higher in infants and children under six years (P = 0.004). Two-thirds of the patients had a parasitaemia between 5,000-40,000/μL, only 5% over 70,000/μL. Gametocyte prevalence at recruitment was similar in both age groups; mean gametocytaemia was significantly lower in patients under six years (P = 0.036).

**Table 1:** Baseline characteristics of enrolled patients, all patients (A) and stratified by age group (B, under five years and over five years)

**A**				
***Baseline characteristics***	**n = 348**	**95% CI**	**range**	
*Mean age (years)*	17.1	15.7-18.5	1-81	
*Female (%)*	40.2	35.0-45.4	-	
*Mean temperature (°C)*	38.1	38.0-38.2	35.6-40.6	
*Geometric mean parasite density*	9,720	8,760-10,785	1,000-99,932	
*Gametocytes present (%)*	9.5	6.4-12.6	-	
*GM gametocyte density*	1,305	899-1,894	40-6,798	
**B**	**<5 years**		**>5 years**	
***Baseline characteristics***	**n = 64**	**95% CI**	**n = 284**	**95% CI**
*Mean age (years)*	3.3	2.9-3.6	20.2	18.7-21.7
*Female (%)*	46.9	34.3-59.4	38.7	33.0-44.4
*Mean temperature (°C)*	38.2	38.0-38.5	38.0	37.9-38.1
*Geometric mean parasite density*	12,046	9,029-16,073^1^	9,262	8,299-10,336
*Gametocytes present (%)*	9.4	2.0-16.7	9.5	6.1-13.0
*GM gametocyte density*	435	82-2,320^2^	1,666	1,250-2,221

^1^ Student’s *t*-test, P = 0.004 for a difference in means between the two age groups.

^2^ Wilcoxon rank sum test, P = 0.036 for a difference in means between the two age groups.

Fifty-seven patients were excluded from the final analysis because PCR results showed mixed infection with *P. vivax* (51) or negative results (six). Re-evaluation of the slides confirmed the molecular results in some cases but sub-microscopic parasitaemia of *P. vivax* was assumed in most mixed infections. Molecularly negative samples showed parasitaemia less than 1,000/μL in microscopy. All cases with mixed infections showed adequate clinical and parasitological response at day 42.

Of the remaining 348 patients, 32 (9.2%) were lost to follow-up during the study (Figure 1). The age was equally distributed among those lost to follow-up and all other study participants. The relation between children ≤5 years and elder patients was similar in both groups but relatively more boys ≤5 years (73.5%) were lost. There was no evidence for a real difference in parasitaemia between those lost to follow-up and the other participants (p>0.05). Those lost to follow-up after day 2 were already negative in blood slides at day 2.

### Study outcome

Until day 42, 286 patients showed an adequate clinical and parasitological response (ACPR). Twenty-eight LTF were observed during follow-up, 16 (4.6%) were LCF and 12 (3.4%) were LPF. PCR-correction showed 16 recrudescences, of which 62.5% were symptomatic, and incidence of 12 new infections. Recrudescences started on day 18 until 42 (mean: 37 days); the first new infection occurred at day 28 (mean: 39 days).

The LTF rate was barely influenced by drop outs as only one was lost to follow-up between day 28 and 42.

For per protocol (PP) analysis, PCR-corrected cure rates at day 28 and 42 were 99.4 (95% CI 98.5-100.0) and 94.7 (95% CI 92.2-97.2), respectively. Uncorrected cure rates at day 28 and 42 were 99.1 (95% CI 98.0-100.0) and 91.1 (95% CI 87.9-94.3), respectively (Table 2). At day 42, PCR-corrected cure rate for children ≤5 years was 90.6 (95% CI 82.4-98.7) compared to 95.6 (95% CI 93.0-98.2) in older patients, but the difference lacked significance (P = 0.17) ( Additional file 1).

**Table 2:** Outcome at day 28 and 42 for all patients

**Treatment outcome**	**Day 28**	**Day 42**
Lost to follow-up (%)	31/348 (8.9)	32/348 (9.2)
Inability to tolerate oral treatment^1^ (%)	2/348 (0.6)	2/348 (0.6)
Early treatment failure (%)	0	0
Late treatment failure (%)	3/348 (0.9)	28/348 (8.0)
Late clinical failure (%)	3/348 (0.9)	16/348 (4.6)
Late parasitological failure	0	12/348 (3.4)
ACPR	312/348 (89.7)	286/348 (82.2)
Infection with different species (%)	0	0
New infection P. falciparum (%)	1/348 (0.3)	12/348 (3.4)
Recrudescences (%)	2/348 (0.6)	16/348 (4.6)
Cure rate per protocol, PCR-uncorrected (%, 95% CI)	312/315 (99.1, 98.0-100.0)	286/314 (91.1, 87.9-94.3)
Cure rate per protocol, PCR-corrected (%, 95% CI)	312/314 (99.4, 98.5-100.0)	286/302 (94.7, 92.2-97.2)
Cure rate intention-to-treat, PCR-uncorrected (%, 95% CI)	312/348 (89.7, 86.4-92.9)	286/348 (82.2, 78.1-86.2)
Cure rate intention-to-treat, PCR-corrected (%, 95% CI)	312/347 (89.9, 86.7-93.1)	286/336 (85.1, 81.3-88.9)

^1^ Those patients defined as “adverse event requiring change in anti-malarial therapy prior to completion of full dose of study drug”. They were included in the intention-to-treat analysis but excluded from the per-protocol analysis.

For the intention to treat analysis (ITT), uncorrected cure rates at day 28 and 42 were 89.7 (95% CI 86.4-92.9) and 82.2 (95% CI 78.1-86.2), respectively; PCR-corrected cure rates at day 28 and 42 were 89.9 (95% CI 86.7-93.1) and 85.1 (95% CI 81.3-88.9), respectively (Table 2).

### Fever clearance

Fever was more rapidly cleared in patients >5 years (Table 3).

**Table 3:** Fever and parasitaemia clearance

***Outcome***	**<=5 years of age**	**>5 years of ag**	**Total**
Fever, n/N (%)			
Day 2	1/60 (1.7)	10/274 (3.7)	11/334 (3.3)
Day 3	0	3/269 (1.1)	3/329 (0.9)
Microscopically determined parasitaemia, n/N (%)			
Day 2	2/60 (3.3)	4/274 (1.4)	6/334 (1.8)
Day3	0	2/270 (0.7)	2/329 (0.6)
Microscopically determined gametocytaemia, n/N (%)			
Day 0	6/64 (9.4)	26/284 (9.5)	33/348 (9.5)
Day 2	5/60 (8.3)	12/274 (4.4)	17/334 (5.1)
Day 3	4/60 (6.7)	6/269 (1.9)	10/329 (3.0)
Day 7	1/57 (1.8)	6/265 (2.3)	7/322 (2.2)
Day 28	1/54 (1.9)	2/259 (0.8)	3/313 (1.0)
Day 42 (37)	1/53 (1.9)	1/258 (0.4)	2/301 (0.7)

### Parasite clearance and associated variables

Six patients (1.9%) showed a prolonged duration till clearance with parasites still present at day 2 (four patients) - mean half-life t_2_ of 9.9 hours [range 7.3-13.9] - and at day 3 (two patients) - mean half-life t_3_ of 21.4 hours [range 11.9-30.9]. All parasites were cleared at day 7. For those without parasitaemia at day 2 or later, mean half-life of <2.9-4.8 hours can be assumed, considering 1,000-100,000 parasite/μL at admission. Values are only an approximation (±8 hours) as it was not possible to take the blood exactly 48 or 72 hours later (Table 3).

Mean age of late responders (>48 hrs) was 11 years [range 1.3-22.0]. They were from all clusters; two were ≤5 years, underweight and stunted. Initial parasitaemia at day 0 was below 10,000/μL (mean parasitaemia 7,058/μL, range 3,800-8,800/μL) in all six patients. Criteria for an early treatment failure, defined by WHO [28], were not fulfilled. Parasitaemia at day 3 was 20% and 2% of day 0, respectively. Both patients with parasites at day 3 showed ACPR at day 42.

However, there was evidence for a strong association of prolonged parasite presence (>48 hrs) with recrudescence (OR 9.5, 95% CI 1.6-57.5, P = 0.01), meaning a recrudescence was 9.5 times more likely when parasites were cleared after day 2 ( Additional file 2).

Parasite density, on the other hand, seemed to have no influence on recrudescent infections. Recrudescences presented with a geometric mean parasitaemia of 8,665/μL (95% CI 6,619-11,344/μL) at day 0. Mean geometric parasitaemia of 286 patients with ACPR was 9,431/μL (95% CI 8,402-10,586/μL). There was no evidence of a real difference between these groups (P = 0.80).

### Microscopic gametocyte clearance and associated variables

Gametocyte prevalence was 9.5% by microscopy. Eighty percent reduction could be observed between day 0 and day 7 ( Additional file 3). Only two patients developed microscopic gametocytaemia after treatment start (Table 3). Three patients had gametocytes on day 28. They were already positive on day 0 and below 13 years (median 7 years).

The association of recrudescences (after day 28) with gametocytaemia at day 28 was significant (OR 38.5, 95% CI 3.1-485.4, P<0.01) ( Additional file 2). There was also evidence for a strong association of prolonged parasite presence with gametocytaemia on day 7 and day 28 (OR 10.1, 95% CI 1.0-99.8; P = 0.05 and OR 20.4, 95% CI 1.4-309.8; P<0.03, respectively). Controlled for site, gender, age and parasite density, the prevalence of gametocytes at any point in time between day 0 to day 42 was more likely in season 1 than in season 2 (OR 0.47, 95% CI 0.22-1.01, P = 0.05).

### Molecular and submicroscopic investigations

Species PCR was done for all samples on day 42 with 12 (4.2%) of the 286 patients with ACPR being positive for *P. falciparum*, no parasites were microscopically found. Therefore, sub-microscopic levels of either asexual or sexual parasitaemia can be assumed.

In 32 randomly chosen microscopically negative samples from day 0, the gametocyte-specific *Pfs25*-mRNA could still be detected in 22 (70%) of the samples by QT-NASBA. That means that microscopy revealed less than one of seven of the prevalence of gametocytes in these 32 patients. As storage duration and conditions of filter-paper samples as well as blood spot sizes were not standardized, molecular quantification of gametocytes was not possible.

### Symptoms, adverse events and pregnancy

Most patients had fever, headache, shivering and nausea at admission. Abdominal pain and diarrhoea were less frequent ( Additional file 4): 2.9% of the patients had adverse events (AE) on day 2 and 3.3% on day 3 with abdominal pain and diarrhoea being the most prevalent ( Additional file 5). Two infants with high parasitaemia (>95,000/μL) were recruited but did not tolerate oral treatment. After re-dosing and repeated vomiting, the infants were referred to the ward for intravenous treatment; one died the same day. Both patients were reported as serious adverse events (SAE) but classified as “adverse event requiring change in anti-malarial therapy prior to completion of full dose of study drug”. A rapid aggravation of symptoms during the enrolment procedure was suspected, no signs of severe malaria were noticed at admission. Both children received IV quinine, as oral therapy was not tolerated.

No further SAE occurred and no pregnancy (exclusion criteria) was reported.

## Discussion

Of all 348 patients, 91.3% responded clinically and parasitically adequately to the treatment until day 42. Adverse events were expectedly mild to moderate with low prevalence. Abdominal pain and diarrhoea were the most frequent symptoms.

The PCR-corrected failure rate showed incidence rates of 3.8% for new infections and 5.1% for recrudescences at day 42. No early treatment failure occurred. Compared to a trial in 2006, these data show a slight increase in recurrent parasitaemia as none had been detected until day 42 [37].

Other Ethiopian studies detected also high cure rates but most had a follow-up period of 28 days only [19-23]. In this study, the PCR-corrected cure rate per protocol at day 28 was 99.4% (95% CI 98.5-100.0). This result emphasizes the need for follow-up periods of at least 42 days. Most recrudescences occurred after day 28. The recrudescence rate in children ≤5 years was surprisingly high with 9.4%. None of the children with recrudescences had diarrhoea or vomiting at admission or during therapy. Apart from limitations discussed in the next paragraph, the weight-adapted drug dose is non-linear but stepwise. Two of the five children with LTF had calculated drug doses of less than 2 mg/kg and 12 mg/kg body weight, respectively. Furthermore, weight-adapted linear or non-linear doses might not correlate with plasma drug levels in children in general.

Unfortunately, there are several limitations concerning this study. Drug levels were not tested and only the first drug intake was observed. As absorption is fat-dependent, fatty food was provided and patients were precisely instructed how and when to take the rest of the medication. However, intake was not directly controlled and absorption individually varies, also impaired by symptoms like diarrhoea [38]. Therefore, recrudescences and also the prolonged parasite presence could be due to insufficient drug levels. The advantage of this study is the closer approach to a real life situation.

Another limitation is the method of genotyping. Differentiation of recrudescences and re-infections by PCR is unprecise, especially in low to moderate transmission areas with a limited diversity of strains [32]. Similar strains, defined as recrusdescence, could be new infections instead. The recrudescence rate given might be lower in reality.

Artemether has a very short half-life of about one hour. Lumefantrine has a half-life of three to six days and is able to clear the remaining parasites and to prevent recurrent parasitaemia [39,40]. The first recrudescence was observed early at day 18. However, due to limitations mentioned above, other reasons than strain resistance might have caused recrudescences. New infections seemed to be efficiently prevented in the first four weeks.

Six patients showed prolonged presence of parasites detectable still at day 2 and day 3, the calculated half-life time was prolonged. As stated above, this could have been caused by low drug levels. Another theory is the hypothesis of a dormant non-responding parasite stage causing delayed susceptibility to drugs [41,42]. This might be responsible for late clearance rates, though genetic analysis revealed a heritable trait at the Cambodia–Thailand border indicating changes in parasite genetics and a potential for spread [15]. Controlled ACT studies with measurement of drug levels and observed drug intake should be conducted in Africa to examine drug efficacy and clearance rates.

The low number of recrudescences and prolonged parasite presence (>48 hrs) impaired multivariate analysis. Risk factors for these outcomes could not be determined. There was strong evidence for an association of both outcomes but the result has to be considered with caution as the sample size is low. However, an association of recrudescence and prolonged parasite presence (72 hrs) was described recently [29]. Parasite clearance data from 18,699 patients with uncomplicated falciparum malaria were analysed. Blood smear result on day 3 was reported as a good predictor of subsequent treatment failure in low- and moderate-transmission areas. The risk of recrudescence was <5% if parasitaemia was cleared before day 3, patients with parasitaemia on day 3 on the other hand had a significantly higher risk in low-transmission areas (12%) and in moderate-transmission areas (34%) to develop recrudescences. Less pronounced but still significant was the association of parasitaemia at day 2 and recrudescences in moderate transmission areas.

This association was observed in the underlying study. Recurrent parasitaemia occurred in two patients with parasites at day 2 (P = 0.01). Prevalence of patients with parasites at day 3 was 0.6% (2/348) only. Even if adequate drug levels could be assumed, tolerance to artemether would not be suspected. Artemisinin resistance seems highly unlikely if prevalence of patients with parasites at day 3 is <3% [29].

Further analysis indicated a correlation of (prolonged) presence of gametocytes with prolonged parasitaemia and recrudescences. Sample size was low as mentioned but associations seemed plausible. Gametocytes have more opportunities to develop if parasite presence is prolonged and sub-microscopic level as with recrudescences seem to suffice for gametocyte production.

Interestingly, gametocytaemia was more frequent in season 2008 than 2009. The broad introduction of ACT in this region might have influenced the gametocyte carriage in the population. ACT clears gametocytes better than S/P, the previous first-line drug. ACT is supposed to destroy the early stages of gametocytes [16,43]. The carrier reservoir might be reduced since introduction of ACT.

A random selection of samples showed very high levels of gametocytaemia with QT-NASBA technique compared to microscopy. Therefore, higher submicroscopic levels have to be assumed on recruitment as well as after treatment. Recent studies showed a difference of factor 3–6 between microscopic and submicroscopic gametocyte prevalence [44,45]. Less than one gametocyte per μL is sufficient to infect a mosquito [43]. Gametocytes detected by microscopy were rapidly reduced under AL in this study but 2.2% of carriers remained positive until day 7. A much higher proportion of submicroscopic gametocytaemia can be assumed. ACT seems to have less or no effect on mature gametocytes [16,43]. Therefore, stage V gametocytes, if already developed at start of therapy, might circulate for two to three weeks. The reservoir is a permanent risk for further transmission and should be measured in clinical trials as well as in population-based studies with recently published methods [35].

Further, an additional drug for gametocyte clearance should be considered in the area. A single dose of primaquine, best given at day 8, might be sufficient for gametocyte clearance [46]. Unfortunately, prevalence of G6PD enzyme deficiency is unknown. As tests are not yet available in this area, the application of primaquine remains ethically problematic, although single-dose treatment seems acceptable [43].

Interestingly, *P. falciparum* mono-infection occurred in only half of the malaria cases in the study area. Prevalence of mixed infections with *P. vivax* and infections with *P. vivax* alone was high and has increased over the last years [[9], pers comm with health centres in Jimma and surrounding]. In Serbo, 70% of the malaria patients were infected with *P. vivax* only. However, there was surprisingly no re-infection with *P. vivax* observed. One explanation for this and the very few re-infections with *P. falciparum* might be that new bed nets were given free of charge by the government to every patient visiting the health centres.

*Plasmodium vivax* infections diagnosed by PCR and afterwards excluded from analysis showed ACPR in all cases, no relapses occurred. Data on *P. vivax* and AL are unavailable in this area. Chloroquine is still first-line treatment of *P. vivax.* A recent study from another area from Ethiopia observed treatment with AL and CQ in vivax malaria. Recurrent parasitaemia rates were 19% and 8%, respectively, although, the evening dose of AL was not observed [47]. Studies from Asia reported higher recurrence rates with AL compared to CQ or dihydro-artemisinin/piperaquine as well [48,49]. However, relapses seem to occur in Ethiopia later than in Asia [43] and might be expected after day 42.

## Conclusion

AL showed good effectiveness in Jimma. The overall PCR-corrected cure rate per protocol was 94.7%, resulting in a recrudescence rate of below 10%. The PCR-corrected per-protocol failure rate for children ≤5 years was 9.4%. Low drug levels due to malabsorption and dosing problems in children could be responsible. To monitor closely the further development of drug susceptibility in this area, a continuous surveillance should be established and controlled ACT studies with measurement of drug levels and observed drug intake should be conducted.

## Competing interests

The authors declare that they have no competing interests.

## Authors’ contributions

TE coordinated the clinical study in Ethiopia, participated in the molecular genetic studies and helped draft the manuscript. NA, KB and SF participated in and carried out the clinical studies. AW and MP established the QT-NASBA technology, carried out the gametocyte analysis and revised the manuscript. TL participated in study design and coordination and drafted the manuscript. NB-R designed the study protocol, was PI of the study, carried out the molecular genetic studies as well as the sequence alignments, conducted the statistical analysis and drafted the manuscript. All authors read and approved the final manuscript.

## Supplementary Material

Additional file 1 **Outcome at day 42 stratified in age groups**. Description: The table shows the outcome at day 42 stratified in the age groups > 5 years and ≤ 5 years. The cure rates are in general lower for the under five years old children but the difference is not significant. Click here for file

Additional file 2 **Association with outcome recrudescence (ACPR & Recrudescences, n = 302)**. Description: The data show a multi-variate analysis with the outcome recrudescences as dependent variable and independent variables like parasitaemia at day 0, gametocytaemia over time, and parasite clearance, controlled for age and gender. Delayed clearance and gametocytaemie at day 28 were associated with recrudescences. There was evidence for a strong association (P ≤ 0.01) but confidence intervals were wide. Click here for file

Additional file 3 **Clearance of microscopically detected gametocytes over time**. Description: Clearance of microscopically detected gametocytes during follow-up in both age groups and overall. Clearance in older patients seemed to be faster than in children below 6 years of age. Click here for file

Additional file 4 **Symptoms at recruitment (n = 348)**. Description: The data show all symptoms presented at admission with absolute and relative frequencies and duration. Fever, headache and shivering were the leading symptoms. Click here for file

Additional file 5 **Adverse events (AE) during follow-up**. Description: The table presents all adverse events reported until day 7. Abdominal pain was the most reported AE. Prevalence was overall low. Click here for file
