# Supplementary material for: Open-label trial with artemether-lumefantrine against uncomplicated Plasmodium falciparum malaria three years after its broad introduction in Jimma Zone, Ethiopia
Source: Malar J. 2012 Jul 23;11:240. doi: 10.1186/1475-2875-11-240 (PMC3438107; doi:10.1186/1475-2875-11-240)
Supplement: Additional file 1 — Outcome at day 42 stratified in age groups. Description: The table shows the outcome at day 42 stratified in the age groups > 5 years and ≤ 5 years. The cure rates are in general lower for the under five years old children but the difference is not significant. [file 1475-2875-11-240-S1.doc]

**Additional file 1**. Outcome at day 42 stratified in age groups

| ***Treatment outcome day 42*** | **≤5 years** | **> 5 years** |
| --- | --- | --- |
| *Lost to follow-up (%)* | 7/64 (11.0) | 25/284 (8.8) |
| *Inability to tolerate oral treatmen^1^ (%)* | 2/64 (3.1) | 0 |
| *Early treatment failure (%)* | 0 | 0 |
| *Late treatment failure (%)* | 7/64 (11.0) | 21/284 (7.4) |
| *Late clinical failure (%)* | 5/64 (7.9) | 11/284 (3.9) |
| *Late parasitological failure* | 2/64 (3.1) | 10/284 (3.5) |
| *ACPR* | 48/64 (75.0) | 238/284 (83.8) |
| *Infection with different species (%)* | 0 | 0 |
| *New infection P. falciparum (%)* | 2/64 (3.1) | 10/284 (3.5) |
| *Recrudescences (%)* | 5/64 (7.9) | 11/284 (3.9) |
| *Cure rate per protocol,*  *PCR-uncorrected (%, 95% CI)* | 48/55 (87.3,  78.2-96.4) | 238/259 (91.9,  88.5-95.2) |
| *Cure rate per protocol,*  *PCR-corrected (%, 95% CI)* | 48/53 (90.6,  82.4-98.7) | 238/249 (95.6,  93.0-98.2)^2^ |
| *Cure rate intention-to-treat,*  *PCR-uncorrected (%, 95% CI)* | 48/64 (75.0,  64.1-85.9) | 238/284 (83.8,  79.5-88.1) |
| *Cure rate intention-to-treat,*  *PCR-corrected (%, 95% CI)* | 48/72 (77.4,  66.7-88.1) | 238/274 (86.9,  82.8-90.9) |

^1^ Those patients defined as “Adverse event requiring change in antimalarial therapy

prior to completion of full dose of study drug”. They were included in the intention-

to-treat analysis but excluded from the per-protocol analysis.

^2^Fisher’s exact test for both age groups, P = 0.17
