# Supplementary material for: Open-label trial with artemether-lumefantrine against uncomplicated Plasmodium falciparum malaria three years after its broad introduction in Jimma Zone, Ethiopia
Source: Malar J. 2012 Jul 23;11:240. doi: 10.1186/1475-2875-11-240 (PMC3438107; doi:10.1186/1475-2875-11-240)
Supplement: Additional file 2 — Association with outcome recrudescence (ACPR & Recrudescences, n = 302). Description: The data show a multi-variate analysis with the outcome recrudescences as dependent variable and independent variables like parasitaemia at day 0, gametocytaemia over time, and parasite clearance, controlled for age and gender. Delayed clearance and gametocytaemie at day 28 were associated with recrudescences. There was evidence for a strong association (P ≤ 0.01) but confidence intervals were wide. [file 1475-2875-11-240-S2.doc]

**Additional file 2. Association with outcome recrudescence (ACPR & Recrudescences, N**=302)

| ***Parameters*** | | **n/N (%)** | | **Recrudescent**  **m/n (%)** | **OR (95% CI)** | | **P-value**^1^ |
| --- | --- | --- | --- | --- | --- | --- | --- |
| *Male* | | 173/302 (57.3) | | 10/173 (5.8) | Baseline | | 0.67 |
| *Female* | | 129/302 (42.7) | | 6/129 (4.7) | 0.8 (0.3-2.3) | |
| *Age groups in years* | | | |  | | | |
| *0-5* | 53/302 (17.6) | | | 5/53 (9.4) | Baseline | | 0.23^2^ |
| *6-15* | 99/302 (32.8) | | | 5/99 (5.1) | 0.5 (0.1-1.9) | |
| *16-30* | 110/302 (36.4) | | | 4/110 (3.6) | 0.4 (0.1-1.4) | |
| *31-50* | 33/302 (10.9) | | | 2/33 (6.1) | 0.6 (0.1-3.4) | |
| *51-81* | 7/302 (2.3) | | | 0 | 0 | |
| *Parasite group, µL^-1^* | | | |  | | | |
| *1000-5000* | | | 77/302 (25.5) | 3/77 (3.9) | | Baseline | 0.53^2^ |
| *5001-10000* | | | 101/302 (33.4) | 8/101 (7.9) | | 2.1 (0.5-8.4) |
| *10001-40000* | | | 99/302 (32.8) | 5/99 (5.1) | | 1.3 (0.3-5.7) |
| *40001-70000* | | | 11/302 (3.6) | 0 | | 0 |
| *70001-100000* | | | 14/302 (4.6) | 0 | | 0 |
| *ACPR*  *Clearance after day 2* | | | 296/302 (98.0)  6/302 (2.0) | 14/296 (4.7)  2/6 (33.3) | | Baseline  9.5 (1.6-57.5) | 0.01 |
| *No microscopic gametocytaemia* | | | 267/302 (89.1) | 14/267 (5.2) | | Baseline |  |
| *Gametocytaemia, day 0* | | | 33/302 (10.9) | 2/33 (6.1) | | 1.2 (0.2-5.5) | 0.86 |
| *Gametocytaemia, day 28* | | | 3/302 (1.0) | 2/3 (66.7) | | 38.5 (3.1-485.4) | <0.01 |

^1^Controlled for gender and/or age, ^2^Test for trend
