# Supplementary figures and images for: Open-label trial with artemether-lumefantrine against uncomplicated Plasmodium falciparum malaria three years after its broad introduction in Jimma Zone, Ethiopia
Source: Malar J. 2012 Jul 23;11:240. doi: 10.1186/1475-2875-11-240 (PMC3438107; doi:10.1186/1475-2875-11-240)

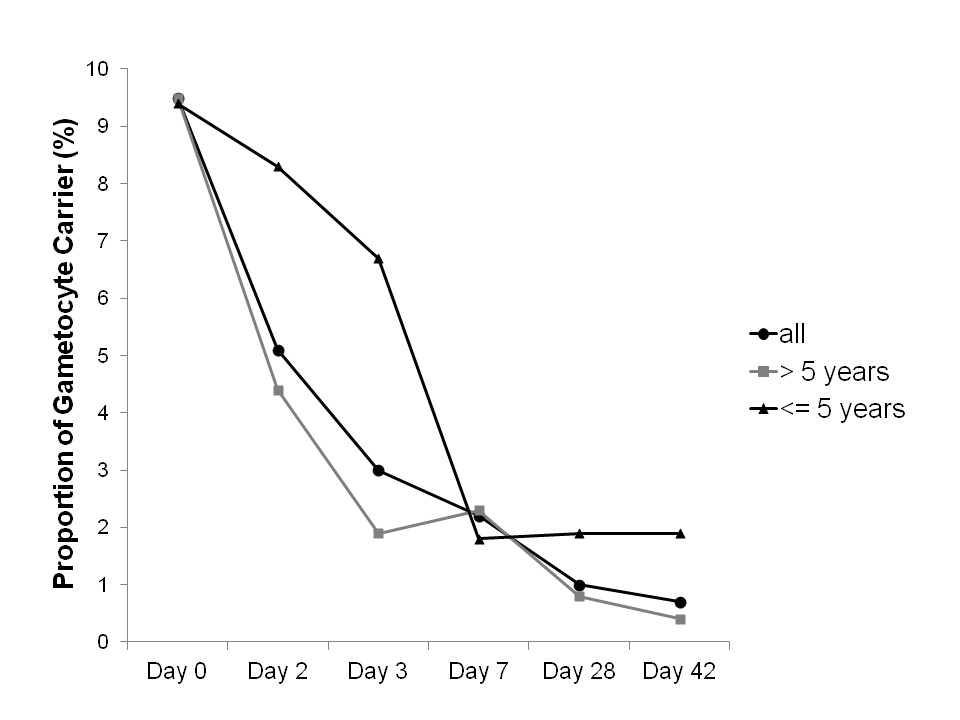

Supplement: Additional file 3 — Clearance of microscopically detected gametocytes over time. Description: Clearance of microscopically detected gametocytes during follow-up in both age groups and overall. Clearance in older patients seemed to be faster than in children below 6 years of age. [file 1475-2875-11-240-S3.tiff]
