# Supplementary material for: Open-label trial with artemether-lumefantrine against uncomplicated Plasmodium falciparum malaria three years after its broad introduction in Jimma Zone, Ethiopia
Source: Malar J. 2012 Jul 23;11:240. doi: 10.1186/1475-2875-11-240 (PMC3438107; doi:10.1186/1475-2875-11-240)
Supplement: Additional file 4 — Symptoms at recruitment (n = 348). Description: The data show all symptoms presented at admission with absolute and relative frequencies and duration. Fever, headache and shivering were the leading symptoms. [file 1475-2875-11-240-S4.doc]

**Additional file 4. Symptoms at recruitment (N=348**)

| ***Symptoms at recruitment*** | **None**  **n (%)** | **1-2 days,**  **n (%)** | **3-5 days,**  **n (%)** | **>6 days,**  **n (%)** | **Any,**  **n (%)** |
| --- | --- | --- | --- | --- | --- |
| *Fever (>38.0°C)* | 1 (0.3) | 258 (74.1) | 81 (23.3) | 8 (2.3) | 347 (99.7) |
| *Headache* | 10 (2.9) | 248 (71.3) | 82 (23.6) | 8 (2.3) | 338 (97.1) |
| *Shivering* | 46 (13.2) | 226 (64.9) | 66 (19.0) | 10 (2.9) | 302 (86.8) |
| *Nausea, Vomiting* | 107 (30.7) | 194 (55.7) | 40 (11.5) | 7 (2.0) | 241 (69.3) |
| *Abdominal Pain* | 261 (75.0) | 68 (19.5) | 19 (5.5) | 0 | 87 (25.0) |
| *Diarrhoea* | 306 (87.9) | 36 (10.3) | 24 (6.9) | 0 | 42 (12.1) |
| *Joint Pain* | 341 (98.0) | 5 (1.4) | 2 (0.6) | 0 | 7 (2.0) |
| *Coughing* | 343 (98.6) | 2 (0.6) | 3 (0.9) | 0 | 5 (1.4) |
