# Supplementary material for: Open-label trial with artemether-lumefantrine against uncomplicated Plasmodium falciparum malaria three years after its broad introduction in Jimma Zone, Ethiopia
Source: Malar J. 2012 Jul 23;11:240. doi: 10.1186/1475-2875-11-240 (PMC3438107; doi:10.1186/1475-2875-11-240)
Supplement: Additional file 5 — Adverse events (AE) during follow-up. Description: The table presents all adverse events reported until day 7. Abdominal pain was the most reported AE. Prevalence was overall low. [file 1475-2875-11-240-S5.doc]

**Additional file 5**. Adverse events (AE) during follow-up

| ***Symptoms***  ***(multiple answers)*** | **Day 2** | **Day 3** | **Day 7** |
| --- | --- | --- | --- |
| *Abdominal pain, n (%)* | 6/341 (1.8) | 5/332 (1.5) | 0 |
| *Diarrhoea, n (%)* | 1/341 (0.3) | 5/332 (1.5) | 0 |
| *Nausea^1^, n (%)* | 1/341 (0.3) | 1/332 (0.3) | 0 |
| *Coughing, n (%)* | 2/341 (0.6) | 1/332 (0.3) | 0 |
| *Headache, n (%)* | 0 | 1/332 (0.3) | 0 |
| *Fever (without parasitaemia), n (%)* | 0 | 1/332 (0.3) | 1/322 (0.3) |
| *Vertigo, n (%)* | 0 | 1/332 (0.3) | 0 |
| *Patients with AE, n (%)* | 10/341 (2.9) | 11/332 (3.3) | 1/322 (0.3) |

^1^Two patients with vomiting at day 0, classified as “Adverse event requiring

change in antimalarial therapy prior to completion of full dose of study drug”.
